# Prognosis of patients with acute exacerbation of combined pulmonary fibrosis and emphysema: a retrospective single-centre study

**DOI:** 10.1186/s12890-020-01185-9

**Published:** 2020-05-20

**Authors:** Yuichi Ikuyama, Atsuhito Ushiki, Makoto Kosaka, Jumpei Akahane, Yuichi Mukai, Taisuke Araki, Yoshiaki Kitaguchi, Kazunari Tateishi, Kazuhisa Urushihata, Masanori Yasuo, Hiroshi Yamamoto, Masayuki Hanaoka

**Affiliations:** First Department of Internal Medicine, Shinshu University School of Medicine, 3-1-1, Asahi Matsumoto, Nagano Prefecture, 390-8621 Japan

**Keywords:** Acute exacerbation, Combined pulmonary fibrosis and emphysema, Idiopathic pulmonary fibrosis, Prognosis, Emphysema

## Abstract

**Background:**

Previous analyses of combined pulmonary fibrosis and emphysema (CPFE) cohorts have provided conflicting data on the survival of patients with CPFE. Therefore, the aim of this study was to investigate the clinical prognosis of acute exacerbations (AE) of CPFE.

**Methods:**

We retrospectively reviewed the medical records of patients who had been treated at the Shinshu University Hospital (Matsumoto, Japan) between 2003 and 2017. We identified 21 patients with AE of CPFE and 41 patients with AE of idiopathic pulmonary fibrosis (IPF) and estimated their prognoses using the Kaplan–Meier method.

**Results:**

Treatment content and respiratory management were not significantly different between the two groups before and after exacerbation. At the time of AE, the median serum Krebs von den Lungen-6 level was significantly lower in the CPFE group (Krebs von den Lungen-6: 966 U/μL; white blood cell count: 8810 /μL) than that in the IPF group (Krebs von den Lungen-6: 2130 U/μL, *p* < 0.001; white blood cells: 10809/μL, *p* = 0.0096). The baseline Gender-Age-Physiology scores were not significantly different between the two groups (CPFE, 4.5 points; IPF, 4.7 points; *p* = 0.58). Kaplan–Meier curves revealed that the survival time after AE for patients with CPFE was longer than that for patients with IPF (*p* < 0.001, log-rank test).

**Conclusions:**

Survival prognoses after AE were significantly better for patients with CPFE than that for those with IPF. Our findings may improve the medical treatment and respiratory management of patients with AE-CPFE.

## Background

Combined pulmonary fibrosis and emphysema (CPFE) is characterised by emphysema in the upper lung and fibrosis in the lower lung on chest high-resolution computed tomography (CT) [1]. Previous analyses of CPFE cohorts have provided conflicting data on the survival implications of emphysema coexisting with fibrosis [2–7]. Similar to patients with idiopathic pulmonary fibrosis (IPF), those with CPFE are also at risk of having episodes of acute exacerbation (AE) of their disease [1]. However, there are limited data available regarding the prognosis of patients with AE-CPFE.

Mejia et al. [7] reported that the overall prognosis in patients who had CPFE with the usual interstitial pneumonia (UIP) pattern was poorer than that of patients with IPF. However, Kurashima et al. [4] reported that the overall prognosis was better for patients with CPFE than that for those with IPF. They showed that 31.8% of patients died because of AE-IPF in contrast to 11.1% who died because of AE-CPFE. The differences in mortality rates between AE-IPF and AE-CPFE may have led to differences in the prognosis between patients with IPF and patients with CPFE. Therefore, we compared differences in the prognosis between patients with AE-CPFE and those with AE-IPF.

## Methods

### Study design

We retrospectively reviewed the medical records of patients with CPFE and IPF, who had been treated at the Shinshu University Hospital (Matsumoto, Japan) between 2003 and 2017. Patients with IPF were included if they met the 2018 clinical practice guideline criteria for IPF, that were established by the American Thoracic Society, European Respiratory Society, Japanese Respiratory Society, and Latin American Thoracic Association [8]. Patients with CPFE were included if they had fibrosis concomitant with UIP, which characterises IPF, because the therapeutic effect in patients with IPF differs from that of other interstitial pneumonias. They were also included if they had a low-attenuation area (LAA) score that was ≥7 points based on chest CT with a slice thickness of < 2.5 mm. The LAA score was calculated as follows: the lungs were divided into six fields (i.e., the upper lobe, middle lobe, and lower lobe of each lung). The upper, middle, and lower lung fields represent the area of the lung above the level of the tracheal carina, the area below the level of the inferior pulmonary vein, and the area between the upper and lower fields, respectively. Emphysema was visually assessed in each patient, and each lung field was assigned a score according to the following criteria established by Goddard et al. [9]: 0 = %LAA < 5%, 1 = %LAA ≥5 and < 25%, 2 = %LAA ≥25 and < 50%, 3 = %LAA ≥50 and < 75%, and 4 = %LAA ≥75%. The scores for each of the six lung fields were summed to obtain the total emphysema score. The CT images were reviewed by two chest radiologists or pulmonologists. All scores were evaluated during the stable period before an AE. Acute exacerbation of IPF was diagnosed based on the diagnostic criteria described by Collard et al. [10] in 2016 as follows: (1) previous or concurrent diagnosis of IPF, (2) acute worsening or development of dyspnoea, typically within a 1-month duration, (3) CT imaging revealed new, bilateral ground-glass opacity and/or consolidation superimposed on a background pattern consistent with the UIP pattern, and (4) respiratory deterioration is not fully explained by cardiac failure or fluid overload. Depending on whether an underlying trigger for AE was determined, the diagnostic criteria categorised AE as ‘triggered AE’ or ‘idiopathic AE’. In this study, only patients with idiopathic AE were included. An acute exacerbation of CPFE was diagnosed using the same criteria for AE-IPF. Patients who met the criteria for any connective tissue disorder were excluded from the study. At the time of an AE, two chest radiologists or pulmonologists classified the patterns on chest CT as a peripheral pattern, multifocal pattern, or diffuse pattern, as described in a previous report [11]. The baseline clinical parameters were obtained within 1 month of the initial diagnosis during a stable status. The survival status was obtained from medical records.

### Statistical analysis

Categorical data are presented as the number and percentage, and continuous data are presented as the median and interquartile range.

Differences in the categorical variables between the two groups were compared using the chi-squared test or the G test, and differences in the continuous variables between the two groups were analysed using the Mann–Whitney *U* test. The prognosis was estimated using the Kaplan–Meier and log-rank tests. The treatment outcome was assessed as death within 30 days and death within 90 days. A *P*-value of < 0.05 was considered statistically significant.

## Results

### Baseline clinical characteristics and data of patients with CPFE and IPF

Based on the selection criteria described previously, the clinical data of 21 patients with AE-CPFE and 41 patients with AE-IPF were enrolled in the present study. One patient with IPF, who had the CT pattern of probable UIP was diagnosed based on surgical lung biopsy findings. The thin-slice CT findings revealed that all other patients with IPF exhibited the UIP pattern. No patient was referred for lung transplantation. Table 1 shows the baseline clinical characteristics and data from patients with IPF and CPFE-UIP before an AE. There were no significant differences in the sex ratio, age, percentage of patients who used corticosteroid or antifibrotic drugs (i.e., pirfenidone or nintedanib), and supplemental oxygen usage between the IPF and CPFE groups. However, cigarette smoking (in pack-years) was significantly higher in the CPFE group than that in the IPF group (*p* = 0.0058).

**Table 1:** Baseline clinical characteristics and data for patients with IPF and patients with CPFE-UIP

Parameter	Patients with IPF (*n* = 41)	Patients with CPFE (*n* = 21)	*p*-value
Sex			
Male/Female	34/7 (83%/17%)	19/2 (91%/9%)	0.676
Age (y)	71 (67–75)	74 (69–76)	0.14
Cigarette smoking (pack-years)	32 (0.875–44.75) (*n* = 38)	51(29.5–69) (*n* = 19)	0.0058
Patients using corticosteroid drugs	8 (19.5%)	6 (28.6%)	0.419
Patients using immunosuppressant drugs	1 (2%)	1 (4.8%)	0.788
Patients using antifibrotic drugs (e.g. pirfenidone or nintedanib)	10 (24.4%)	3 (14.3%)	0.552
Patients receiving NAC inhalation	1 (0.2%)	0(0%)	0.73
Patients receiving supplemental oxygen	12 (29.3%)	9 (42.9%)	0.285
LAA score (points)	1 (0–3)	10 (9–15)	< 0.001
%FVC (%)	64.5 (53.2–80.9) (*n* = 28)	79.3 (73.7–90.1) (*n* = 17)	0.0069
%DLco (%)	39.8 (29.8–51.9) (*n* = 21)	35.4 (30.6–41.1) (*n* = 14)	0.2461
FEV_1_ / FVC	84.75(83–88.4)	81.2(72.3–87.5)	0.018
GAP score (points)	4.5 (4–6) (*n* = 22)	4.5 (4–5) (*n* = 14)	0.58
CPI	55.4 (45.4–61.8) (*n* = 22)	52.6 (46.3–58.0) (*n* = 14)	0.41
P/F ratio (Torr)	331 (291–358) (*n* = 26)	328 (315–366) (*n* = 17)	0.555
TR-PG (mmHg)	22.3 (15.5–31.2) (*n* = 15)	22.6 (18.1–26.1) (*n* = 6)	0.969
WBC count (/mm^3^)	7805 (6575–8337.5) (*n* = 33)	6780 (6270–8000) (*n* = 20)	0.238
CRP (mg/dL)	0.14 (0.06–0.26) (*n* = 29)	0.11 (0.04–0.26) (*n* = 19)	0.51
LDH (IU/L)	222 (201–267) (*n* = 29)	234 (207–252) (*n* = 19)	0.78
KL-6 (U/mL)	1246 (899–1494) (*n* = 33)	636.5 (442–1102) (*n* = 19)	0.004
RF (IU/mL) < 10 /≥10	25/11 (*n* = 36) a	9/8 (*n* = 17)^b^	0.242
ANA < 1:40 / ≥1:40	20/15 (*n* = 35) ^c^	9/9 (*n* = 18) ^d^	0.621

The parameters are expressed as number (percentage) or median (interquartile range). For insufficient data, the confirmed numbers are indicated by ‘*n* =’

*IPF* idiopathic pulmonary fibrosis, *CPFE* combined pulmonary fibrosis and emphysema, *UIP* usual interstitial pneumonia, *NAC* n-acetyl-cysteine, *LAA* low attenuation area, *%FVC* percent predicted forced vital capacity, *%DLco* diffusing capacity for carbon monoxide, *FEV1/FVC* forced expiratory volume in 1 s /forced volume vital capacity ratio, *GAP* Gender-Age-Physiology, *CPI* composite physiologic index, *P/F* ratio of partial pressure arterial oxygen to fraction of inspired oxygen (PaO2/FiO2, *TR-PG* transtricuspid pressure gradient, *WBC* white blood cell, *CRP* C-reactive protein, *LDH* lactate dehydrogenase, *KL-6* Krebs von den Lungen-6, *RF* rheumatoid factor, *ANA* anti-nuclear antibodies

^a^14 out of 36 patients were data at acute exacerbation. ^b^ 8 out of 17 patients were data at acute exacerbation. ^c^ 13 out of 35 patients were data at acute exacerbation. ^d^10 out of 18 patients were data at acute exacerbation

The percentage of predicted forced vital capacity (%FVC) was significantly higher in the CPFE group than that in the IPF group (*p* = 0.0069). There was no significant difference in the percentage of predicted carbon monoxide diffusing capacity between the two groups. The forced expiratory volume in 1 s/forced volume vital capacity (i.e., FEV_1_/FVC) ratio was significantly lower in the CPFE group than that in the IPF group (*p* = 0.018). There were no significant differences in the Gender-Age-Physiology (GAP) score, ratio of partial pressure arterial oxygen to fraction of inspired oxygen (PaO2/FiO2 [i.e., P/F ratio]), composite physiologic index (CPI) score, white blood cell counts, C-reactive protein levels, and lactate dehydrogenase levels between the two groups. Serum levels of Krebs von den Lungen-6 (KL-6) were significantly higher in the IPF group than in the CPFE group (*p* = 0.004).

Echocardiography was performed in 16 patients in the IPF group and 7 in the CPFE group. Among them, the transtricuspid pressure gradient (TR-PG) could be measured in 15 patients and 6 patients, respectively. There was no significant difference in the TR-PG between the two groups.

There were no significant differences between the two groups of patients who were positive for rheumatoid factor and anti-nuclear antibodies. No patients had no physical findings suggestive of collagen disease (e.g. joint pain, rash, or Raynaud’s symptoms).

There were no significant differences between the groups in terms of the history of cardiovascular disease (4 cases in IPF, 4 cases in CPFE; *p* = 0.52), malignant tumors (5 cases in IPF, 6 cases in CPFE; *p* = 0.21), diabetes (8 cases in IPF, 5 cases in CPFE; *p* = 0.95), and apoplexia cerebri (2 cases in IPF, 1 case in CPFE; *P* = 0.55).

### Laboratory data of patients with CPFE and IPF at the time of the AE

Table 2 shows the laboratory data of the two groups at the time of the AE. The C-reactive protein level, lactate dehydrogenase level, and P/F ratio were not significantly different between the two groups at the time of the AE. However, the serum KL-6 levels and white blood cell counts were significantly lower in the CPFE group than those in the IPF group at the time of the AE (*p* < 0.001 and *p* = 0.0096, respectively). The CT pattern was not significantly different between the two groups at the time of the AE.

**Table 2:** Laboratory data at the time of acute exacerbation in patients with IPF and CPFE-UIP

Parameter	Patients with IPF (*n* = 41)	Patients with CPFE (*n* = 21)	*p*-value
WBC count (/mm^3^)	10,030 (8620–12,500)	88,810 (6460–9320)	0.0096
CRP (mg/dL)	5.5 (2.4–10.7)	5.85 (2.68–7.15)	0.466
LDH (IU/L)	352 (261–453)	289 (263–349)	0.151
KL-6 (U/mL)	1596 (1225–2525)	966 (429–1310)	< 0.001
P/F ratio (Torr)	204 (148–277)	204 (130–261)	0.417
TR-PG (mmHg)	38.0 (33.0–48.1) (*n* = 5)	27.8 (25.4–55.0) (*n* = 7)	0.871
CT pattern: peripheral or multifocal/diffuse	27/14 (65.9%/34.1%)	17/4 (89.0%/ 19.0%)	0.345

The data are expressed as median (interquartile range) or number (percentage)

*IPF* idiopathic pulmonary fibrosis, *CPFE* combined pulmonary fibrosis and emphysema, *WBC* white blood cell, *CRP* C-reactive protein, *LDH* lactate dehydrogenase, *KL-6* Krebs von den Lungen-6, *P/F ratio* ratio of partial pressure arterial oxygen to fraction of inspired oxygen (PaO2/FiO2), *TR-PG* transtricuspid pressure gradient, *CT* computed tomography

Echocardiography was performed in 6 patients in the IPF group and 7 in the CPFE group at the time of acute exacerbation. Among them, TR-PG was measured in 5 and 7 patients, respectively. Although the number of cases was small, there was no significant difference in the TR-PG between the two groups.

### Treatment for AE in the two groups

The treatments for AE in the two groups are shown in Table 3. All patients in both groups were administered corticosteroids. There were no significant differences in terms of respiratory care, duration of positive-pressure ventilation, or length of stay in the intensive care unit between the two groups. Patients with IPF were more likely to undergo treatment with immunosuppressants than those with CPFE.

**Table 3:** Treatment for acute exacerbation in patients with IPF and CPFE-UIP

Treatment	Patients with IPF (*n* = 41)	Patients with CPFE (*n* = 21)	*p*-value
Number of patients using corticosteroids	41 (100%)	21 (100%)	–
Number of patients of using immunosuppressants	19 (46.3%)	4 (19.0%)	0.08
CY	14 (34.1%)	3 (14.3%)	
CyA	4 (9.7%)	2 (9.5%)	
TAC	1 (2.4%)	0 (0%)	
Number of patients receiving respiratory care			0.927
IPPV	8 (19.5%)	3 (14.3%)	
NPPV	13 (31.7%)	7 (33.3%)	
HFT	3 (7.3%)	1 (4.8%)	
Only O_2_	17 (41.5%)	10 (47.6%)	
Duration of positive-pressure ventilation (days)	9.5 (3.75–39.25) (*n* = 20)	5(3.25–7.75) (*n* = 10)	0.628
ICU stay (days)	10 (4–17.) (*n* = 13)	4.5 (3.25–8) (*n* = 6)	0.122

*Note*: Most invasive respiratory management methods are listed

The data are expressed as the number (percentage) or as the median (interquartile range)

*CPFE* combined pulmonary fibrosis and emphysema, *CY* cyclophosphamide, *CyA* cyclosporine, *HFT* high-flow therapy, *ICU* intensive care unit, *IPF* idiopathic pulmonary fibrosis, *IPPV* invasive positive-pressure ventilation, *NPPV* noninvasive positive-pressure ventilation, *O*_*2*_ oxygen, *TAC* tacrolimus

### Prognosis of the two groups

**Fig. 1:**
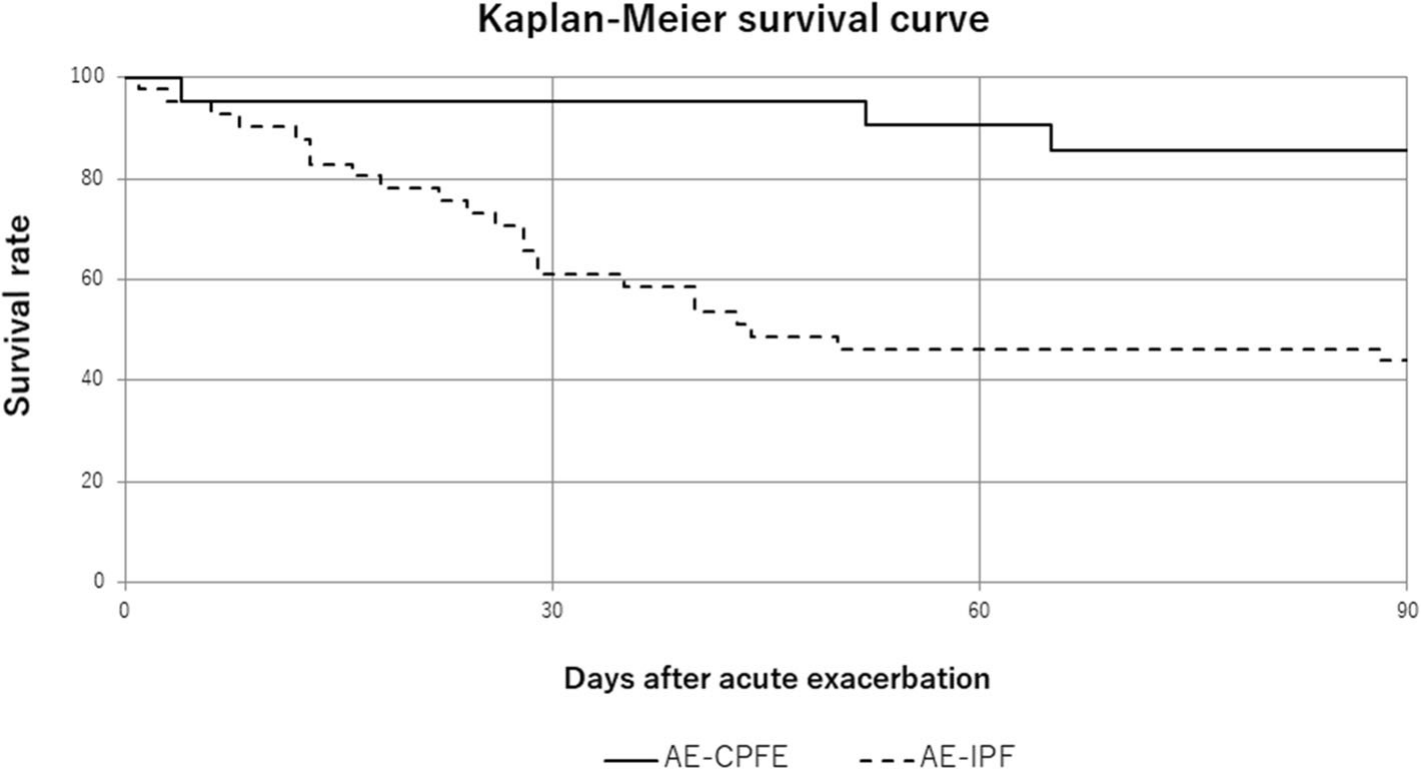
Survival of patients with AE-CPFE and AE-IPF based on the Kaplan–Meier method. The survival rate of patients with AE-CPFE (*n* = 21; solid line) is significantly lower than that of patients with AE-IPF (*n* = 41; dashed line) (*P* < 0.001, log-rank test). The 30- and 90-day survival rates of patients in the AE-CPFE group are 95.2 and 85.7%, respectively. The 30- and 90-day survival rates of patients in the AE-IPF group were 61.0 and 43.9%, respectively (30 days: *p* = 0.0066; 90 days: *p* = 0.0039). Abbreviations: AE-CPFE, acute exacerbation of combined pulmonary fibrosis and emphysema; AE-IPF, acute exacerbation of idiopathic pulmonary fibrosis

The Kaplan–Meier plot in Fig. 1 shows the survival rate of patients with AE-CPFE (solid line) and AE-IPF (dashed line). All patients died of respiratory failure.due to AE. The survival rate was significantly higher in patients with AE-CPFE than in patients with AE-IPF (*p* < 0.01, log-rank test). The 30- and 90-day survival rates for patients in the AE-CPFE group were 95.2%) and 85.7%, respectively; these values were significantly higher than those (61.0 and 43.9%, respectively) for patients in the AE-IPF group (30 days: *p* = 0.0066, 90 days: *p* = 0.0039). Among the patients who died within 90 days, 13 in the AE-IPF group and 2 in the AE-CPFE group had the peripheral pattern, and 10 patients in the AE-IPF group and 1 in the AE-CPFE group had the multifocal/diffuse pattern (*p* = 0.345).

## Discussion

In this retrospective study, we compared the clinical prognosis of patients with AE-CPFE with that of patients with AE-IPF. Our results demonstrated that the prognosis of AE-CPFE was significantly better than that of AE-IPF.

CPFE is characterised by emphysema in the upper lung and pulmonary fibrosis in the lower lung on CT images. Usual interstitial pneumonia and nonspecific interstitial pneumonia are typically observed in patients with CPF. However, we only compared cases of IPF with cases of CPFE with findings of fibrosis concomitant with UIP on thin-slice chest CT images.

In the present study, patients with AE-CPFE had a significantly better prognosis and a higher survival rate than those with AE-IPF. A recent study demonstrated that the survival rate of AE-IPF was 66% within 1 month and 41% within 3 months [12]. This finding was consistent with the findings of our study, which showed a survival rate of 61.0% within 30 days and 43.9% within 90 days in patients with AE-IPF.

Previous studies have reported that a low %FVC and percentage of predicted carbon monoxide diffusing capacity in lung function tests and high KL-6 and lactate dehydrogenase serum levels before AE are risk factors, that contribute to a poor prognosis in patients with AE-IPF [13, 14]. The present results also demonstrated that the %FVC was significantly lower and the levels of KL-6 were significantly higher in the IPF group than in the CPFE group before an AE.

There were no significant differences in the P/F ratio, GAP score, CPI and use of supplemental oxygen between the two groups before AE. This finding suggested that the respiratory status of the patients in the two groups was similar before AE. Thus, the disease severity before AE did not differ between the two groups.

Patients with IPF were more likely to undergo treatment with immunosuppressants than those with CPFE. After acute exacerbations, immunosuppressants were added to steroids in cases of poor response. This demonstrated that the clinical course of AE-IPF was worse than that of AE-CPFE.

Yoon et al. [15] reported that IPF developed concomitantly with emphysema in some lung fields in 42% of their patients, and this finding was based on high-resolution CT. In the present study, patients with CPFE had an LAA score ≥ 7, which implied that the lung field with emphysema was larger and that with fibrosis was smaller in the CPFE group, than the lung field with emphysema and the lung field with fibrosis in the IPF group.

The KL-6 level at the time of AE was significantly higher in patients with IPF than that in those with CPFE. This finding suggests that the lung injury in patients with IPF was more severe than that in those with CPFE. The area of diffuse alveolar damage, which is a histopathological hallmark of AE-IPF, ​​may be smaller in AE-CPFE than in AE-IPF, because of emphysema. We propose that the difference in the composition of fibrosis in the lung fields between the two groups may have led to the difference in prognosis between patients with AE-IPF and patients with AE-CPFE.

One report [11] indicated that the mortality rate of patients with AE-IPF was higher for individuals with the diffuse ground-glass pattern than for those with the multifocal pattern or peripheral pattern on CT images. The present study revealed that there were no significant differences in the distribution of the CT pattern classifications between the two groups. Moreover, the prognosis of the CPFE group was good, even in patients with the diffuse pattern type. This finding suggests that the pathogenic mechanism of AE-CPFE may differ from that of AE-IPF.

Another possibility is that the pathogenesis of emphysema may antagonise the pathogenesis of ongoing diffuse alveolar damage. Wang et al. [16] demonstrated that urokinase plasminogen activator (uPA) and the urokinase plasminogen activator receptor (uPAR) were overexpressed in alveolar macrophages and in the bronchial epithelium in patients with chronic obstructive pulmonary disease. Moreover, another study [17] demonstrated that lung fibrin clearance was accelerated in a lung-specific inducible uPA-expressing transgenic mouse line. This transgenic mouse line was protected from fibrosis and mortality associated with bleomycin-induced lung injury. Furthermore, the risk of radiation pneumonitis was lower in patients with severe emphysema, such as the patients in our study, than in patients with no underlying lung disease [18]. However, this is a hypothesis, and we intend to test this hypothesis in future studies.

Our study had several limitations. First, it consisted of a single-centre retrospective study, that included a small sample of patients. Some patients were transferred to our hospital after the onset of the AE; thus, their data before the AE were insufficient. Second, only one IPF patient had undergone a surgical lung biopsy, and patients with UIP on high-resolution CT may not necessarily exhibit UIP in pathological examination. However, the patients’ clinical characteristics, physiological impairments, and clinical courses were entirely compatible with IPF. Third, only some patients underwent bronchoscopy, and the bronchoalveolar lavage fluid could not be evaluated at the time of the AE. Therefore, the pathogenic mechanisms underlying the differences between AE-CPFE and AE-IPF should be analysed in future research.

## Conclusions

In conclusion, we found that the survival prognosis of AE-CPFE was significantly better than that of AE-IPF for the first time. The differences in the mortality rates between patients with AE-IPF and AE-CPFE may have led to the difference in the prognosis between patients with IPF and those with CPFE. Our findings may improve the medical treatment and respiratory management of patients with AE-CPFE.

## Data Availability

Not applicable.

## Abbreviations

AE: Acute exacerbation
CPFE: Combined pulmonary fibrosis and emphysema
CT: Computed tomography
FVC: Forced vital capacity
GAP: Gender-Age-Physiology
IPF: Idiopathic pulmonary fibrosis
LAA: Low-attenuation area
LDH: Lactate dehydrogenase
TR-PG: Transtricuspid pressure gradient
UIP: Usual interstitial pneumonia
uPA: Urokinase plasminogen activator
uPAR: Urokinase plasminogen activator receptor
